# The Influence of Reorientational and Vibrational Dynamics on the Mg^2+^ Conductivity in Mg(BH_4_)_2_·CH_3_NH_2_

**DOI:** 10.1021/acs.chemmater.4c01947

**Published:** 2024-09-16

**Authors:** Mads B. Amdisen, Yongqiang Cheng, Niina Jalarvo, Daniel Pajerowski, Craig M. Brown, Torben R. Jensen, Mikael S. Andersson

**Affiliations:** † Interdisciplinary Nanoscience Center (iNANO) and Department of Chemistry, University of Århus, Langelandsgade 140, Århus C DK-8000, Denmark; ‡ Neutron Scattering Division, Oak Ridge National Laboratory, Oak Ridge, Tennessee 37831, United States; § NIST Center for Neutron Research, National Institute of Standards and Technology, Gaithersburg, Maryland 20899, United States; ∥ Department of Chemistry - Ångström Laboratory, Uppsala University, Box 538, Uppsala SE-751 21, Sweden

## Abstract

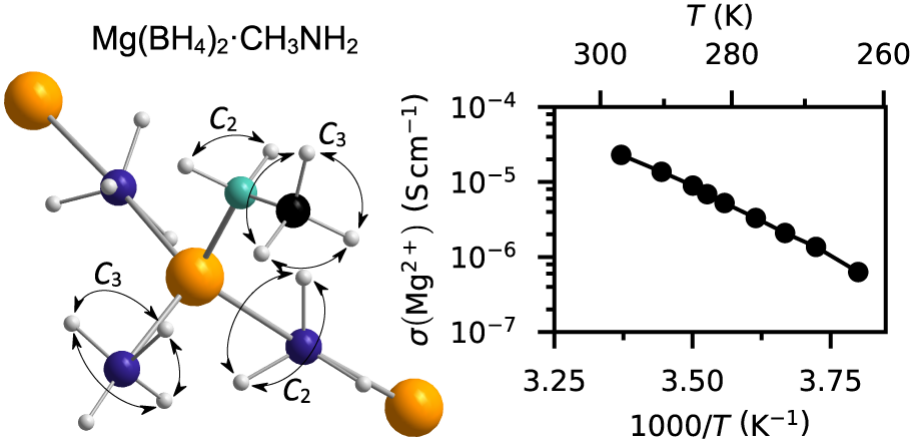

Reorientational dynamics in solid electrolytes can significantly enhance the ionic conductivity, and understanding these dynamics can facilitate the rational design of improved solid electrolytes. Additionally, recent investigations on metal hydridoborate-based solid electrolytes have shown that the addition of a neutral ligand can also have a positive effect on the ionic conductivity. In this study, we investigate the dynamics in monomethylamine magnesium borohydride (Mg(BH_4_)_2_·CH_3_NH_2_) with quasielastic and inelastic neutron scattering, density functional theory calculations, and molecular dynamics simulations. The results suggest that the addition of methylamine significantly speeds up the reorientational frequency of the BH_4_^–^ anion compared to Mg(BH_4_)_2_. This is likely part of the explanation for the high Mg-ion transport observed for Mg(BH_4_)_2_·CH_3_NH_2_. Furthermore, while the dynamics of both the BH_4_^–^ anion and the CH_3_ group of the methylamine ligand is rapid, the NH_2_ group of the methylamine ligand exhibits much slower reorientations, as confirmed by both experimental and computational investigations. Notably, molecular dynamics calculations reveal mean square displacements of 0.387 Å^2^ for NH_2_, 1.503 Å^2^ for CH_3_, and 1.856 Å^2^ for BH_4_^–^ using a trajectory of 10 ps. This study confirms the simultaneous presence of fast dynamics and high ionic conductivity in a metal borohydride-based system and can function as an experimental foundation for future studies on dynamics in hydrogen-rich solid electrolytes.

## 1 Introduction

The demand for rechargeable batteries with high energy densities is rapidly increasing along with the growing demand for electric vehicles and sustainable energy storage solutions. Solid-state batteries offer a potential solution to this challenge by enabling the use of metallic anodes and more efficient cell stacking.^1^ The utilization of solid-state electrolytes (SSEs) can also improve battery safety due to higher mechanical stability and lower flammability compared with the organic electrolytes used in conventional lithium-ion batteries. Because of the low crustal abundance of lithium and its tendency to form dendrites, alternative anode materials are desired, and magnesium serves as a promising candidate. Multiple magnesium borohydride-based SSEs show compatibility with metallic magnesium and display the highest ionic conductivities among magnesium ionic conductors near room temperature.^2−7^ Recently, a Mg-TiS_2_ battery with the composite material Mg(BH_4_)_2_·1.6NH_3_–MgO as SSE was proven to be the best-performing inorganic solid-state magnesium battery so far, thereby progressing toward a greater use of metal borohydride-based SSEs.^8^ Because the SSE in most cases is the primary bottleneck of solid-state battery development, determining the fundamental properties of the best electrolytes can provide invaluable information for the rational design of future, improved systems.

Investigations on the dynamics in various magnesium borohydride-based systems suggest different functions of the dynamics. A comparison between γ-Mg(BH_4_)_2_ and amorphous Mg(BH_4_)_2_ showed improved ionic conductivity in amorphous Mg(BH_4_)_2_, attributed to a higher fraction of activated rotations of BH_4_^–^ anions, that is, the “paddle-wheel” mechanism, determined from quasielastic neutron scattering (QENS).^9^ A similar effect was observed in Mg(BH_4_)_2_-diglyme_0.5_ with one BH_4_^–^ population performing fast *C*_2_/*C*_3_ motions associated with high ionic conductivity, and one slowly rotating BH_4_^–^ population suggested to improve the thermal stability of the compound. The large chelating diglyme ligand has a solvating effect and primarily exhibits segmental motions in its planar geometry while coordinated to Mg^2+^, in addition to reorientational motion of the end methyl groups.^10^ In a similar manner, addition of water molecules to MgB_12_H_12_ and ZnB_12_H_12_, that is, hydration, has been found to increase the ionic conductivity as compared to their anhydrous counterparts.^7^ Neutral ligands have proven to generally be highly influential on the ionic conductivity of metal borohydrides, and particularly, ammine and alkyl ammine metal borohydrides have been reported to have record high ionic conductivities, attributed to flexible structures held together by dihydrogen bonds and weak dispersion interactions. Computational studies of the compounds LiBH_4_NH_3_^11^ and Mg(BH_4_)_2_·NH_3_^12^ show ligand-assisted migration of interstitial cations, where BH_4_^–^ and NH_3_ move back and forth between the interstitial and framework cations, thereby promoting the cationic migration.

Here, we investigate the dynamics in monomethylamine magnesium borohydride, Mg(BH_4_)_2_·CH_3_NH_2_, with quasielastic and inelastic neutron scattering, density functional theory calculations, and molecular dynamics simulations. We also present the magnesium ionic conductivity of Mg(BH_4_)_2_·CH_3_NH_2_ in the temperature range of 263–298 K (−10 to 25 ^◦^C) and discuss the ionic conductivity in relation to the structural model suggested Mg^2+^ migration pathway and the dynamics of different functional groups.

## 2 Methods

### 2.1 Synthesis

Magnesium borohydride was synthesized according to the protocol described in literature.^13^ For selective isotopic enrichment of the samples, dimethylsulfide-borane (DMS-BH_3_) with ^11^B and with hydrogen H or deuterium D was used, that is, DMS-^11^BH_3_ or DMS-^11^BD_3_. Three different selectively deuterated hexamethylamine magnesium borohydride samples were prepared by solid–gas reactions as described in the literature using the reactants α-Mg(^11^BH_4_)_2_ or α-Mg(^11^BD_4_)_2_ and dry methylamine gas with or without deuteration, that is, CH_3_NH_2_ (>98%, anhydrous, Sigma-Aldrich) or CH_3_ND_2_ (>98%, anhydrous, Eurisotop).^4,14^ The products were Mg(^11^BH_4_)_2_·6CH_3_NH_2_, Mg(^11^BD_4_)_2_·6CH_3_NH_2_, and Mg(^11^BD_4_)_2_·6CH_3_ND_2_. Subsequently, stoichiometric amounts of magnesium borohydride and hexamethylamine magnesium borohydride were mechanochemically treated to produce the compositions Mg(^11^BH_4_)_2_·CH_3_NH_2_, Mg(^11^BD_4_)_2_·CH_3_NH_2_, and Mg(^11^BD_4_)_2_·CH_3_ND_2_. All compositions were verified by powder X-ray diffraction using a Rigaku SmartLab diffractometer equipped with a rotating Cu anode, λ = 1.5604 Å or Co anode, λ = 1.7902 Å. All samples were packed in borosilicate capillaries with a diameter of 0.5 mm and sealed with a vacuum grease. For some of the samples, it was only possible to pack a small amount of sample, resulting in diffraction patterns with low intensities. Rietveld refinements are provided in Figures S1–S6.

Proton impurities on the deuterated moieties were determined from ^1^H nuclear magnetic resonance (NMR) spectroscopy, see Figure S7. The measurements were performed on a Bruker Ascend 400 MHz spectrometer equipped with a ^1^H–^13^C–^15^N 5 mm TXI liquid-state probe. The samples were dissolved in deuterated dimethyl sulfoxide in NMR tubes before measuring. The data were processed and analyzed using MNova software from Mestrelab Research using automatic phase and baseline correction. For Mg(^11^BD_4_)_2_·CH_3_NH_2_, the proton contamination on the borohydride groups was determined to be ∼4.1%, that is, Mg(^11^BD_3.83_H_0.17_)_2_·CH_3_NH_2_, see Table S1. For Mg(^11^BD_4_)_2_·CH_3_ND_2_, the proton contamination on the borohydride groups was determined to be ∼5.6% and ∼10.5% for the amine moieties, that is, Mg(^11^BD_3.77_H_0.23_)_2_·CH_3_ND_1.79_H_0.21_ (Table S1).

### 2.2 Synchrotron Radiation Powder X-ray Diffraction

In situ synchrotron radiation powder X-ray diffraction data were collected at the Diamond Light Source at beamline I11 using λ = 0.826927 Å. The samples were packed in borosilicate capillaries with an inner diameter of 0.5 mm and sealed with vacuum grease. The temperature was regulated with a cryostream.

### 2.3 Quasielastic Neutron Scattering

The quasielastic neutron scattering (QENS) measurements were performed using BASIS^15^ and CNCS^16^ at the Spallation Neutron Source (SNS) in the USA. On BASIS, two different analyzer configurations (Si111 and Si311) were used, which have different maximum energy windows (±100 μeV and ±740 μeV), energy resolutions (3.5 μeV and 15 μeV fwhm), and *Q*-ranges (0.2 to 2.0 Å^–1^ and 0.4 to 3.8 Å^–1^). On CNCS, the incident neutron energy *E*_i_ was 5.6 meV which has a corresponding energy resolution of 118 μeV fwhm and *Q*-range of 0.1 to 3 Å^–1^. The powder samples were evenly distributed in individual aluminum foil pouches with approximately 100–300 mg of powder in each pouch. The pouches were wrapped in an annular shape and placed inside individual annular aluminum cans, which were sealed by using indium O-rings. The entire sample preparation was performed under an inert He atmosphere to avoid decomposition of the sample.

The obtained scattering from a QENS measurement can be described by the scattering function:
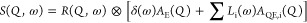
1where *E* = *ℏω* is the neutron energy transfer, *ℏ* is the Planck constant/(2π), ω is the angular frequency, δ is a delta function, *L*_i_ is the Lorentzian function used to describe the quasielastic scattering, *A*_E_ and *A*_QE,i_ are the areas corresponding to the respective delta and Lorentzian functions, and *R*(*Q,*ω) is the instrument resolution function. The QENS data were fitted with the above presented scattering (eq 1) together with a linear background using PAN, which is part of the DAVE distribution.^17^

### 2.4 Inelastic Neutron Scattering

Inelastic neutron scattering experiments were performed at the VISION vibrational spectrometer at the Spallation Neutron Source (SNS) in the USA. Data were collected at 5 K for 1 h for monomethylamine magnesium borohydride with different deuterations, that is, Mg(^11^BH_4_)_2_·CH_3_NH_2_, Mg(^11^BD_4_)_2_·CH_3_NH_2_, and Mg(^11^BD_4_)_2_·CH_3_ND_2_. The instrument allowed an energy range of −2 to 1000 meV with a resolution of 1–1.5%. Subsequent data reduction was done using the analysis cluster at SNS.

### 2.5 Computational Investigations

Modeling by density functional theory (DFT) was performed using the Vienna Ab initio Simulation Package (VASP).^18^ The calculation used the projector-augmented wave (PAW) method^19,20^ to describe the effects of core electrons and the Perdew–Burke–Ernzerhof (PBE)^21^ implementation of the generalized gradient approximation (GGA) for the exchange-correlation functional. The energy cutoff was 800 eV for the plane-wave basis of valence electrons. The total energy tolerance for electronic energy minimization was 10^–8^ eV, and for structure optimization, it was 10^–7^ eV. The maximum interatomic force after relaxation was below 0.005 eV/Å. For crystal simulations, the lattice parameters and atomic coordinates from literature^4^ were used as the initial structure. The optB86b-vdW functional^22^ for dispersion corrections was applied. The vibrational eigenfrequencies and modes were then calculated by solving the force constants and dynamical matrix using Phonopy.^23^ The OCLIMAX software^24^ was used to convert the DFT-calculated phonon results to the simulated INS spectra. To directly calculate the energy barrier associated with individual methyl group rotation, the climbing image nudged elastic band (cNEB) method^25^ was used. Seven images were introduced, with the starting and ending images being the two equilibrium positions of the methyl rotor separated by a relative rotation of 120^◦^.

### 2.6 Electrochemical Impedance Spectroscopy

Electrochemical impedance spectroscopy (EIS) measurements were performed from 1 × 10^7^ to 1 Hz using a Biologic MTZ-35 impedance analyzer with a symmetrical molybdenum sample holder equipped with a 4-probe setup. In an argon-filled glovebox, the samples were pressed into 5 mm diameter pellets in a hydraulic press at ∼1 GPa at RT for 30 s and subsequently placed in the sample holder. A small force was applied by electrically isolated springs to ensure contact between the electrodes and the pellet. The sample holder was sealed before being extracted from the glovebox. The sample holder containing the pellet was cooled and heated in a custom-made setup.

## 3 Results and Discussion

### 3.1 Quasielastic Neutron Scattering

#### 3.1.1 Elastic and Inelastic Fixed Window Scans

To probe the dynamics of Mg(BD_4_)_2_·CH_3_ND_2_, Mg(BD_4_)_2_·CH_3_NH_2_, and Mg(BH_4_)_2_·CH_3_NH_2_, several QENS spectra were measured during heating ∼20–295 K. From these spectra, the elastic and inelastic fixed window scan (EFWS and IFWS) intensities were extracted by integrating selected energy slices of the spectra for each temperature (EFWS −3.5 to 3.5 μeV, IFWS 7 to 14 μeV). In an EFWS experiment, a small energy slice (±3.5 μeV) centered around the elastic peak is integrated for each temperature. Upon heating, the intensity will initially decrease almost linearly due to the Debye–Waller factor. However, at the onset of the dynamics on the instrumental time scale, the slope will change and the intensity will decrease more rapidly upon further heating. As the dynamics become more rapid, the EFWS intensity decreases further until the dynamics become too rapid to be detected on the instrument time scales, leading to a flattening of the EFWS slope. For an IFWS experiment, the energy slice (7 to 14 μeV) is shifted away from the elastic peak. Upon heating, the quasielastic component broadens; that is, some of the intensities are shifted outward away from the elastic peak and will thus result in an increase in the IFWS intensity as intensity enters the energy slice (7 to 14 μeV). As the dynamics become faster, the width of the quasielastic component will continue to increase leading to a decrease in the IFWS intensity as more and more of the quasielastic intensities are situated outside the energy slice. A more detailed description of EFWS and IFWS can be found in ref.^26^

**Figure 1:**
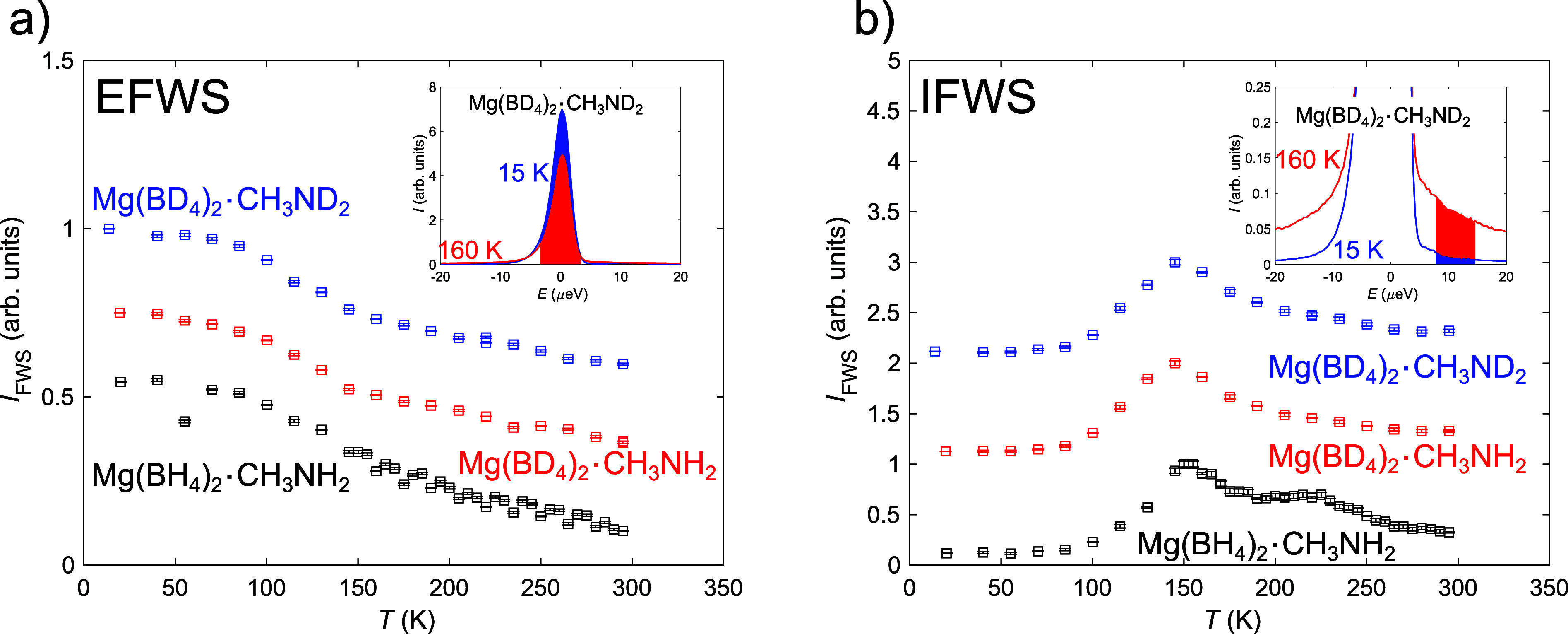
(a) Elastic and (b) inelastic fixed window scans (EFWS and IFWS) for Mg(BD_4_)_2_·CH_3_ND_2_ (blue), Mg(BD_4_)_2_·CH_3_NH_2_ (red), and Mg(BH_4_)_2_·CH_3_NH_2_ (black). The inset in (a,b) shows the integrated region of the QENS spectra, which yields the *I*_FWS_ shown in (a,b), respectively.

In Figure 1a, the EFWS for Mg(BD_4_)_2_·CH_3_ND_2_, Mg(BD_4_)_2_·CH_3_NH_2_, and Mg(BH_4_)_2_·CH_3_NH_2_ is shown. The EFWS starts to decrease around 70 K for all samples indicating the onset of dynamics on the instrument time scale. There are three known crystallographic phases of Mg(BH_4_)_2_·CH_3_NH_2_, that is, α-, α′-, and β-Mg(BH_4_)_2_·CH_3_NH_2_.^4^ The decreasing EFWS correlates well with the initiation of a slow phase transition from the low-temperature phase α-Mg(BH_4_)_2_·CH_3_NH_2_ to the intermediate and high-temperature phases α′-Mg(BH_4_)_2_·CH_3_NH_2_ and β-Mg(BH_4_)_2_·CH_3_NH_2_ from ∼ 80 K as described in ref.^4^ and as shown with synchrotron radiation powder X-ray diffraction data in Figure S8. Since this onset is observed for all of the samples, the dynamics are assigned to CH_3_, as the onset is independent of the amount of deuteration of the samples. Furthermore, the similarities between the EFWS curves for Mg(BD_4_)_2_·CH_3_ND_2_ and Mg(BD_4_)_2_·CH_3_NH_2_ indicate very similar dynamics in the probed temperature range, suggesting that NH_2_ plays a minor role in the studied temperature range. Comparing the slope of the EFWS curves for the three samples, a slight difference in the slope for Mg(BH_4_)_2_·CH_3_NH_2_ can be observed above 160 K. This could indicate a second dynamical onset for Mg(BH_4_)_2_·CH_3_NH_2_ leading to a further decrease in the EFWS intensity as compared to Mg(BD_4_)_2_·CH_3_ND_2_ and Mg(BD_4_)_2_·CH_3_NH_2_. Changes in the dynamics are often better elucidated using the IFWS, which is shown in Figure 1b. In the IFWS, the onset of dynamics is observed as a significant increase in the IFWS intensity, which occurs at around 70 K for all samples, consistent with the observations in the EFWS. Above 150 K, the IFWS intensity starts to decrease indicating that some of the dynamically active species start exiting the instrumental time scale (set by the IFWS energy slice); that is, they become too fast to probe using the selected IFWS energy slice. However, it is clear that for all samples, some dynamically active species exist all the way to 295 K, suggesting a distribution of onset temperatures for CH_3_ dynamics. For Mg(BH_4_)_2_·CH_3_NH_2_, a second feature appears at ∼ 200 K, which we identify as the onset of BH_4_^–^ dynamics. The IFWS data for the deuterated samples also suggest a minor presence of the second feature at ∼200 K, which can be attributed to the hydrogen contamination of the BD_4_ group of ∼4.1% and ∼5.6% for Mg(BD_4_)_2_·CH_3_NH_2_ and Mg(BD_4_)_2_·CH_3_ND_2_, respectively.

#### 3.1.2 General Overview of the QENS Spectra

**Figure 2:**
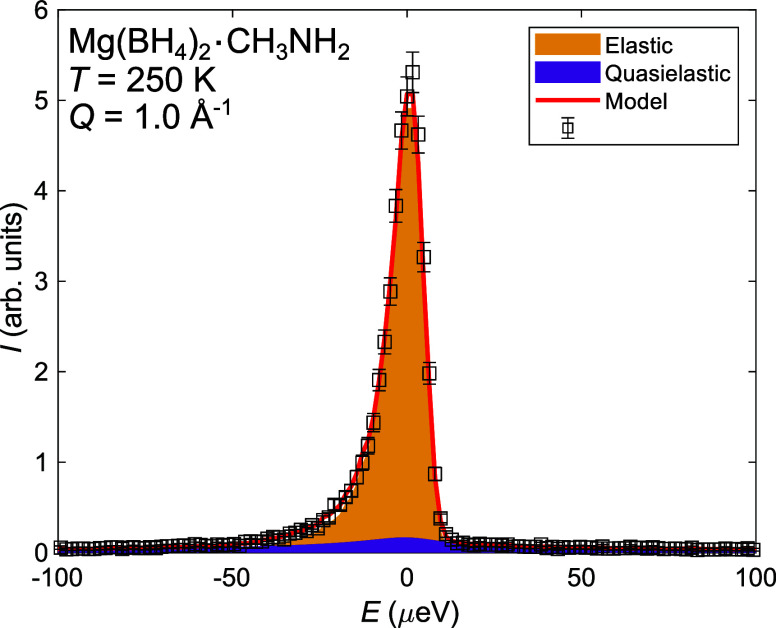
QENS spectra and corresponding fits to *S*(*Q*,ω) at *Q* = 1 Å^–1^ and *T* = 250 K.

The QENS spectra were fitted using eq 1. The lowest temperature (∼20 K) was used as a resolution function since all dynamics had frozen out at this temperature. An example of the data and corresponding fits is shown in Figure 2. As indicated by the EFWS and IFWS, all samples exhibit active dynamics from about 70 K. At this temperature, all samples can be described by a single Lorentzian, while at 100 K, two Lorentzians are needed, indicating that a second set of dynamics enters the instrument time scales.

**Figure 3:**
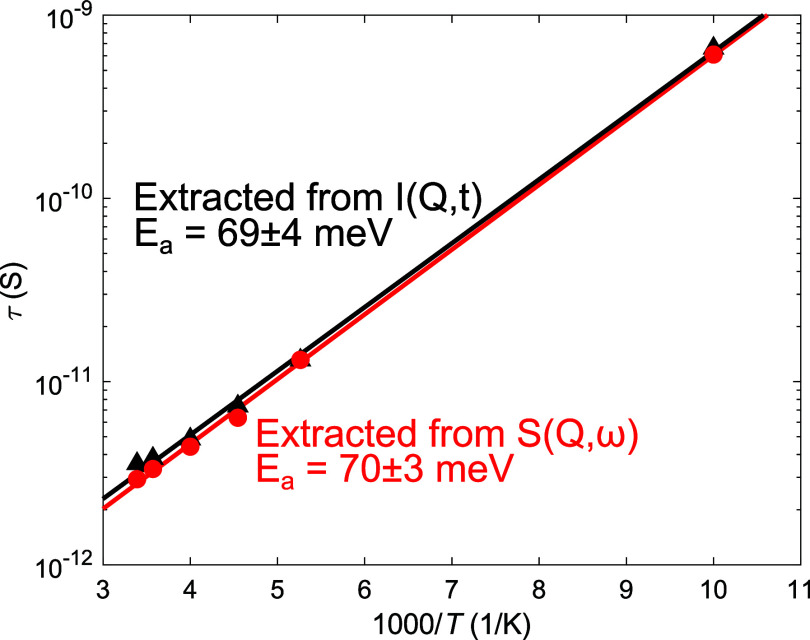
Arrhenius plot of the relaxation time for the CH_3_ group relaxation time τ, extracted from fits of the QENS data from both *S*(*Q*,ω) (energy domain) and *I*(*Q*,*t*) (time domain) for Mg(BD_4_)_2_·CH_3_NH_2_. The solid lines are fits of the temperature dependence of τ to the Arrhenius equation.

At both 70 and 100 K, all samples can be described well with the same Lorentzian widths, indicating that the dynamics are related to CH_3_ reorientations. Upon further heating, more dynamical components enter the instrument time scale for all samples. Since this behavior is present in Mg(BD_4_)_2_·CH_3_ND_2_, where mostly CH_3_ dynamics should be visible, it indicates that there are multiple local environments for CH_3_ leading to multiple CH_3_ reorientational frequencies. This could be due to the presence of two crystalline phases, α′-Mg(BH_4_)_2_·CH_3_NH_2_ and β-Mg(BH_4_)_2_·CH_3_NH_2_, in the temperature range ∼80 to 155 K as described in ref.^4^ The width of the Lorentzian components exhibits no clear dependence on *Q* as shown in Figures S12 and S13. This is indicative of local dynamics, for example, reorientational dynamics, rather than long-range diffusion. These findings are in good agreement with a previous NMR study of Mg(BH_4_)_2_,^27^ which concludes that the dynamics of the BH_4_^–^ anion is of local character. While all three samples can be described using the same set of Lorentzian widths at the same temperature, it becomes apparent that at above 200 K, the ratio between the amplitudes of the Lorentzians is no longer the same for all samples. For Mg(BH_4_)_2_·CH_3_NH_2_, at 220 K, the amplitude of the most narrow Lorentzian increases by about a factor of 2 suggesting that some of the BH_4_^–^ anions are now undergoing reorientations with similar frequencies as some of the CH_3_ groups. To estimate the activation energy for the CH_3_ reorientations, we extracted the width of the strongest and widest Lorentzian as a function of temperature from the QENS spectra of Mg(BD_4_)_2_·CH_3_NH_2_. The width was converted into a relaxation time τ, that is, the average time between reorientational jumps, using the relation , where Γ is the Lorentzian full width at half-maximum and *ℏ* is the reduced Planck constant. The relaxation times are shown in Figure 3 together with a fit to the Arrhenius equation: , where *E*_a_ is the activation energy, τ_0_ is a prefactor, and *k*_B_ is the Boltzmann constant. From the fit, an activation energy of 70 ± 3 meV was determined for the CH_3_ group reorientations. To better take into account the distribution of relaxation times, the data were Fourier transformed from *S*(*Q*,ω) (energy domain) to *I*(*Q*,*t*) (time domain) and fitted using a stretch exponential, which is commonly referred to as a Kohlrausch–Williams–Watts (KWW) function, . Here, *t* is the time, *I* the intensity, *I*_0_ the intensity at *t* = 0, < τ > the average relaxation time of the distribution, and β the stretching exponent, which is related to the distribution of relaxation times. The data were adequately fitted using a stretching exponent of 0.94, indicating that the distribution is relatively narrow, see Figure S11. From the fits, the average activation energy of the dynamics can be extracted by fitting the relaxation times to , which gives an average activation energy of 69 ± 4 meV. Determination of the activation energy for the BH_4_^–^ anion reorientations is not possible from the measured data due to overlap with the CH_3_ dynamics, and the NH_2_ group reorientations are too slow to determine. As a comparison to the experimentally determined energy barrier, DFT calculations were performed using VASP with the cNEB method yielding an energy barrier for the CH_3_ group of ∼74 meV, see Figure S9. The close agreement between the experimentally determined and the calculated energy barrier suggests that the local environment of the CH_3_ group is well-described by the structural model determined in ref.^4^ While it was not possible to determine the reorientational energy barrier of the BH_4_^–^ ion with QENS, the cNEB method suggests an energy barrier of ∼70 meV, see Figure S9. Comparing the activation energy of 70 meV for the CH_3_ group reorientations in Mg(BH_4_)_2_·CH_3_NH_2_ found here to the 48 meV found for CH_3_ group reorientations in CH_3_NH_3_PbI_3_,^28,29^ it can be concluded that the CH_3_ group in Mg(BH_4_)_2_·CH_3_NH_2_ is more hindered than in CH_3_NH_3_PbI_3_. This is likely due to an increased interaction between the CH_3_ group and its surrounding environment such as the NH_2_ group within the CH_3_NH_2_ ligand and other CH_3_NH_2_ ligands.

#### 3.1.3 Elastic Incoherent Structure Factor

**Figure 4:**
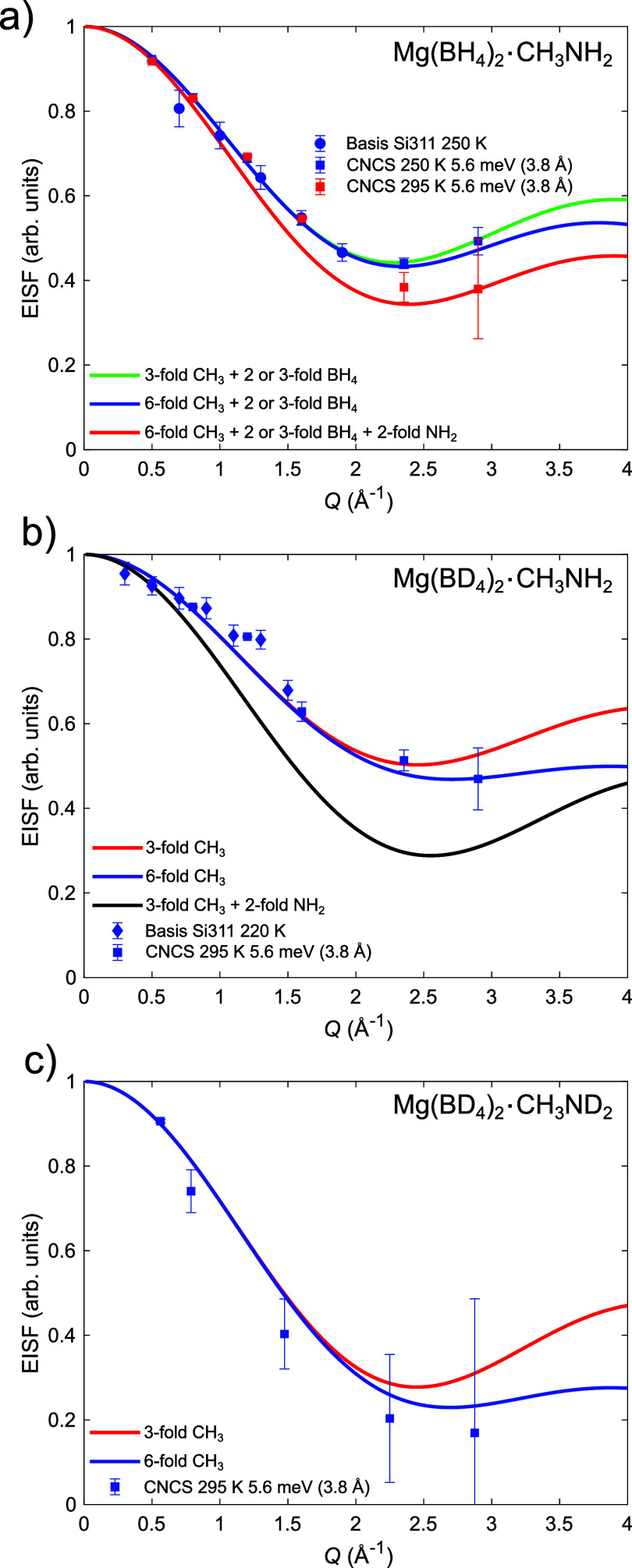
EISF as a function of *Q* for (a) Mg(BH_4_)_2_·CH_3_NH_2_, (b) Mg(BD_4_)_2_·CH_3_NH_2_, and (c) Mg(BD_4_)_2_·CH_3_ND_2_. Error bars correspond to 2 standard deviations. In (a,b), the regions with Al Bragg peaks (∼2.7 and ∼3.1 Å^–1^) have been omitted, while in (c), larger regions of *Q* have been omitted due to Bragg peaks from the sample and the Al sample can.

The elastic incoherent structure factor (EISF) was extracted from the fits of the QENS spectra described in the previous section using the relation:
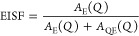
2where *A*_E_(*Q*) and *A*_QE_(*Q*) are the total elastic and quasielastic integrated scattering intensities for a specific value of *Q*. The position and depth of the minimum of the EISF are dependent on the reorientational jump distance and the ratio of atoms undergoing reorientation. The experimentally determined EISFs are shown in Figure 4 together with calculated EISF models for different reorientational motions; see Supporting Information for further information about the EISF models.

**Figure 5:**
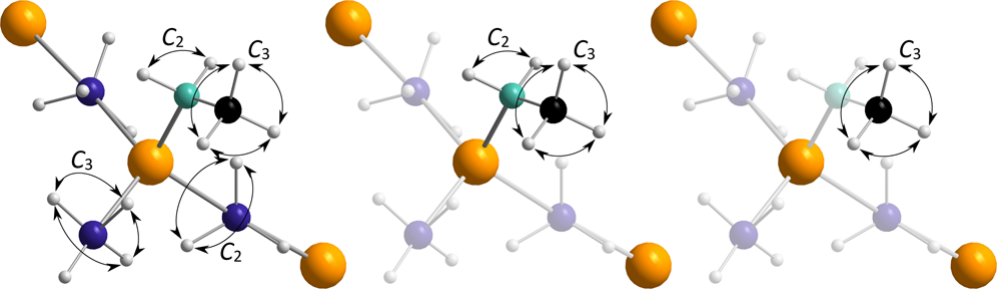
Mg-BH_4_-Mg chains with terminal BH_4_^–^ anions and CH_3_NH_2_ ligands in Mg(BH_4_)_2_·CH_3_NH_2_.^4^ Some of the possible *C*_2_ and *C*_3_ rotations are depicted. The detectable hydrogen dynamics is depicted with increasing deuterium content from left to right. Color scheme: Mg^2+^ (orange), B (blue), N (cyan), C (black), and H/D (gray/light gray).

In the fully protonated sample, Mg(BH_4_)_2_·CH_3_NH_2_, dynamics of all three dynamical species, that is, CH_3_, NH_2_, and BH_4_^–^, is expected to be present. See Figure 5 for a depiction of the various detectable dynamics with varying deuteration. At 250 K, the experimental EISF is best described by a model which takes into account both 3- or 6-fold CH_3_ group and 2- or 3-fold BH_4_^–^ anion reorientations as well as the two static hydrogen of the NH_2_ group. For the BH_4_^–^ anion, 2-fold and 3-fold reorientational motions have the same EISF and are therefore indistinguishable.^26,30^ The total model at 250 K thus consists of a sum of the individual EISFs for CH_3_ group and BH_4_^–^ anion reorientations weighted by the number of H atoms, , since the combined incoherent scattering from B, C, and N is less than 0.1% of the total incoherent scattering. The data thus suggest that the NH_2_ group performs reorientations on a slower time scale than what the spectrometer can capture, since the data do not agree with a model that accounts for NH_2_ group reorientations. This is in good agreement with the findings from the EFWS and IFWS data, which suggest that the NH_2_ group does not exhibit reorientational dynamics in the studied temperature range (∼20–295 K). In Mg(BH_4_)_2_·CH_3_NH_2_, 50% of the BH_4_^–^ anions are located between two Mg^2+^ cations with two of the four hydrogen facing each of the Mg^2+^ cations; that is, these BH_4_^–^ anions act as bridging ions, see Figure 5. This makes a 3-fold reorientation unlikely since it would require the bonds between the BH_4_^–^ anion and the Mg^2+^ cations to be broken. However, a 2-fold reorientation around the Mg-BH_4_-Mg axis can occur without breaking the bonds and is, therefore, more likely. The remaining 50% of the BH_4_^–^ anions act as a terminal ion and only coordinate to one Mg^2+^ cation, where three of the four hydrogens are expected to face the cation making a 3-fold reorientation around the *C*_3_ axis more likely. The QENS data together with previous crystallographic studies^4^ thus suggest that some of the BH_4_^–^ anions perform 2-fold reorientations around one of their *C*_2_ axes, while the remaining anions perform 3-fold rotations around one of their *C*_3_ axes. Similar BH_4_^–^ dynamics has been observed in other metal borohydrides such as Y(BH_4_)_3_·*x*NH_3_ (*x* = 0, 3, 7)^31,32^ and *M*BH_4_ (*M* = Li, Na, K),^33^ emphasizing the influence of the dimensionality of the coordination network in the crystal structure. Since the onset of dynamics for the BH_4_^–^ anions occurs in a narrow temperature range, it is likely that the reorientational frequencies of these 2-fold and 3-fold reorientational motions are very similar.

As mentioned in the synthesis section, there is a hydrogen impurity of about 4% in the BD_4_^–^ anion for Mg(BD_4_)_2_·CH_3_NH_2_. In Mg(BD_4_)_2_·CH_3_NH_2_, the hydrogen impurity of the BD_4_^–^ anions accounts for about 10% of the total incoherent scattering of the sample, while the NH_2_ and CH_3_ groups account for about 36% and 54%, respectively. As seen for Mg(BD_4_)_2_·CH_3_NH_2_ in Figure 4b, the experimental EISFs determined from data collected at 220 and 295 K (blue data points) agree well with an EISF model, which takes into account 3- or 6-fold CH_3_ group reorientations (54%), static NH_2_ groups (36%), and 2- or 3-fold BD(H)_4_^–^ reorientations (10%). While the experimental data agree best with a model with 6-fold CH_3_ group reorientations, the data do not exclude the possibility of 3-fold CH_3_ group reorientations. The findings are in good agreement with the FWS data, which suggest that CH_3_ group reorientations dominate at these temperatures for Mg(BD_4_)_2_·CH_3_NH_2_.

Similar to Mg(BD_4_)_2_·CH_3_NH_2_, a small hydrogen impurity in the BD_4_^–^ anion (∼6%) and in the ND_2_ group (∼10%) in Mg(BD_4_)_2_·CH_3_ND_2_ is present. This means that the BD_4_^–^ anion (including hydrogen impurities) constitutes about 16.5% of the total incoherent scattering, while the ND_2_ (including proton impurities) and the CH_3_ groups constitute about 6.5% and 77% of the total incoherent scattering, respectively. The EISF model that exhibits the best agreement with the data at 295 K is the model that encompasses 6-fold CH_3_ group reorientations and a static ND_2_ group as well as 2- or 3-fold BD_4_^–^ anion reorientations. However, the comparison is limited by the few number of points that can be extracted for the experimental EISF due to the strong Bragg reflections in the *Q* range of ∼1–2 Å ^–1^ which are enhanced in Mg(BD_4_)_2_·CH_3_ND_2_ due to the much larger coherent scattering cross section of D as compared to H. Similar to Mg(BH_4_)_2_·CH_3_NH_2_ and Mg(BD_4_)_2_·CH_3_NH_2_, the QENS data do not definitively exclude the possibility of 3-fold CH_3_ group reorientations instead of the 6-fold reorientations.

To further validate the reorientational frequency of the CH_3_ and NH_2_ groups as well as the BH_4_^–^ anion, a DFT molecular dynamics simulation was performed at 300 K. From the simulations, the mean square displacement (MSD) of the CH_3_ and NH_2_ groups and the anion was determined using a trajectory of 10 ps yielding values of 0.387 Å^2^ for NH_2_, 1.503 Å^2^ for CH_3_, and 1.856 Å^2^ for BH_4_^–^. A larger value of the MSD suggests a higher reorientational mobility, that is, a higher reorientational frequency. The lower MSD for NH_2_ is thus in excellent agreement with the experimental observations presented above.

### 3.2 Inelastic Neutron Scattering

**Figure 6:**
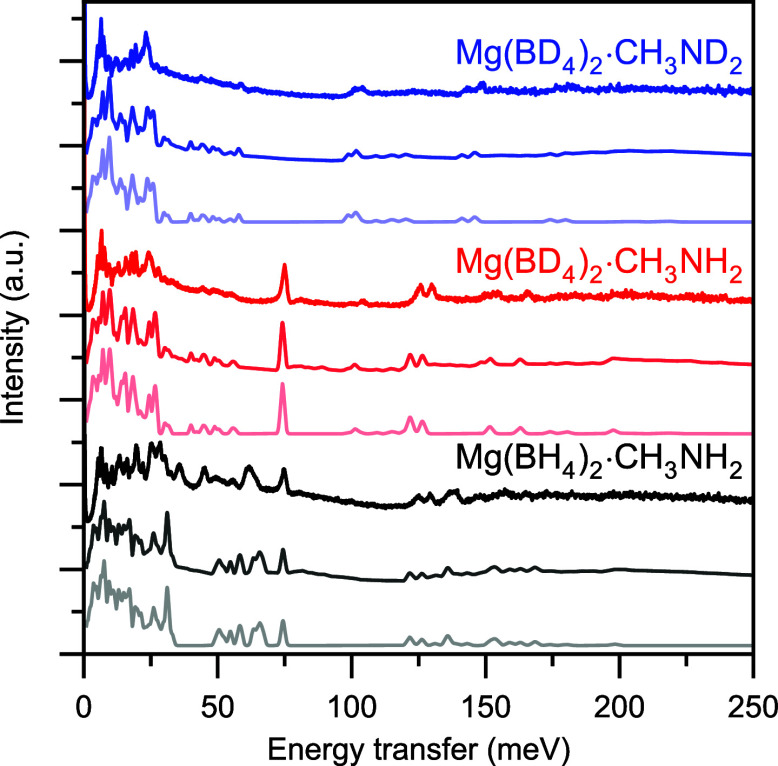
Inelastic neutron scattering spectra of monomethylamine magnesium borohydride with different selective deuterations. Simulated spectra are shown in lighter colors below each experimental spectrum, with the bottom simulated spectrum being the fundamental phonon excitations and the top simulated spectrum being the total phonon excitations.

Inelastic neutron scattering experiments were conducted for monomethylamine magnesium borohydride with different deuterations, see Figure 6. The spectra shown in Figure 6 were collected at 5 K and are compared with simulated spectra of the fundamental and total phonon excitations. The experimental and simulated spectra are generally in good agreement; however, some discrepancies are also observed. The simulated spectra of monomethylamine magnesium borohydride were calculated from the low-temperature α-Mg(BH_4_)_2_·CH_3_NH_2_ (*P*1) structure, which was solved from X-ray diffraction data at 80 K,^4^ and subsequently DFT optimized, see Table S2. This may give rise to some discrepancies due to potential structural inconsistencies at lower temperatures. Additional discrepancies, particularly below ∼50 meV, in all spectra arise from anharmonicity in the experimental spectra, as the simulated spectra are harmonic. Major peaks can be assigned based on the models and the spectra from the differently deuterated samples. Libration around the Mg–N bond is found at ∼7–10 meV, primarily determined from the motion of the CH_3_ group. Vibrational modes involving CH_3_ torsion are found in a relatively broad band around ∼15–20 meV. This roughly corresponds to an energy barrier of 50–80 meV, which is in good agreement with both the QENS data and the DFT calculations. These librations and torsions are the most pronounced features in the spectrum of Mg(BD_4_)_2_·CH_3_ND_2_. The weaker signals in the regions ∼40–60 meV, ∼95–105 meV, and ∼140–150 meV are primarily associated with various bending modes in the CH_3_ND_2_ molecule.

There is a high resemblance between the INS spectra of Mg(BD_4_)_2_·CH_3_ND_2_ and Mg(BD_4_)_2_·CH_3_NH_2_ below an energy transfer of 62 meV due to the NH_2_ group being significantly more static than the CH_3_ group. The most significant differences are observed in the regions ∼72–77 meV and ∼120–133 meV. The region ∼72–77 meV is associated with NH_2_ torsion, however, as the QENS and MD calculations suggest, more extensive motions or rotations of the NH_2_ group are unlikely. The region ∼120–133 meV is mostly associated with bending modes in the CH_3_NH_2_ molecule, similar to the higher energy transfer regions in Mg(BD_4_)_2_·CH_3_ND_2_.

For the fully protonated sample, Mg(BH_4_)_2_·CH_3_NH_2_, the wide region ∼3–31 meV is associated with librations of both the CH_3_NH_2_ ligand and the BH_4_^–^ ion, with both the terminal and bridging anions being active. Large BH_4_^–^ librations are also found in the region ∼45–66 meV. The significant contributions of the BH_4_^–^ ions to the spectrum are also evident from the relative decrease in intensity of the peak at ∼72–77 meV associated with NH_2_ torsion. Minor differences in the spectra are observed at higher energy transfers, most apparent in the region ∼135–140 meV, which is associated with torsion of the bridging BH_4_^–^.

The various libration and torsion modes determined from INS and DFT show a flexible structure with substantial hydrogen dynamics. Additionally, the results are in line with our observations from both the QENS data and the DFT MD and cNEB calculations, which show fast BH_4_^–^ and CH_3_ dynamics and slow NH_2_ dynamics.

### 3.3 Ionic Conductivity

**Figure 7:**
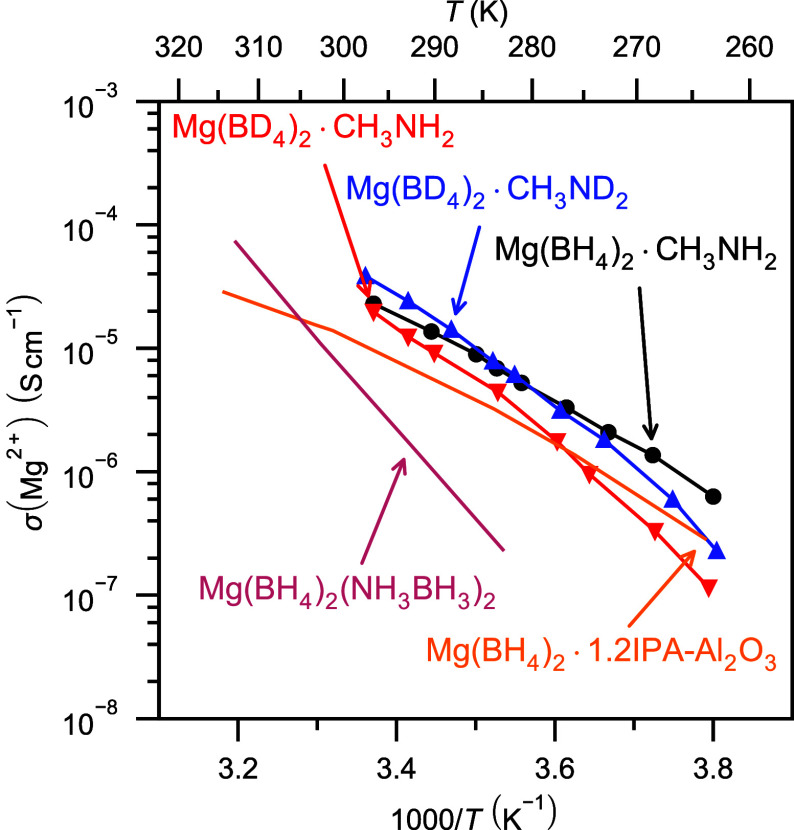
Magnesium ionic conductivities of Mg(BH_4_)_2_·CH_3_NH_2_ (black, circles), Mg(BD_4_)_2_·CH_3_NH_2_ (red, down triangles), and Mg(BD_4_)_2_·CH_3_ND_2_ (blue, up triangles) as well as previously reported low-temperature magnesium solid-state electrolytes for reference.^5,34^

The highest previously reported magnesium ionic conductivities below room temperature are  S cm^–1^ at 283 K (10 ^◦^C) in Mg(BH_4_)_2_(NH_3_BH_3_)_2_ and  S cm^–1^ at 263 K (−10 ^◦^C) in Mg(BH_4_)_2_·1.2(CH_3_)_2_CHNH_2_–Al_2_O_3_(50 wt %).^5,34^ In comparison, monomethylamine magnesium borohydride has ionic conductivities of  S cm^–1^ at 283 K (10 ^◦^C) and  S cm^–1^ at 263 K (−10 ^◦^C), see Figure 7, and consequently has the highest Mg^2+^ ionic conductivities reported at these temperatures. The ionic conductivity of monomethylamine magnesium borohydride has previously been shown to decrease over time. Because the ionic conductivities of the three differently deuterated samples were measured after long-term storage, ∼1 year, the room temperature ionic conductivities are close to the previously reported conductivity of  S cm^–1^ measured 77 days after synthesis and are consequently approximately 1 order of magnitude lower than the reported ionic conductivity measured immediately after synthesis.^4^

Based on the results from QENS and the computational investigations, it is suggested that the fast dynamics of both the BH_4_^–^ anion and the CH_3_ group facilitates a high ionic conductivity, whereas the almost static NH_2_ is unlikely to contribute to Mg^2+^ mobility. This is a notable contrast to the suggested ion conduction mechanism of Mg(BH_4_)_2_·NH_3_, where highly flexible ammonia ligands interchange between interstitial and crystallographic Mg^2+^.^12^ An ion conduction mechanism similar to the one suggested in Mg(BH_4_)_2_·NH_3_ is unlikely in Mg(BH_4_)_2_·CH_3_NH_2_, owing to the larger size of the CH_3_NH_2_ ligand as well as the almost static NH_2_ moiety. This is confirmed by the lack of NH_2_ dynamics in the temperature range investigated with QENS. While it was not possible to determine the activation energy of the BH_4_^–^ 2- and 3-fold rotations, both QENS and MD simulations suggest fast dynamics. Due to the greater charge on the BH_4_^–^ anion compared to the CH_3_ group, a stronger interaction with interstitial Mg^2+^ is also expected, which also suggests that BH_4_^–^ dynamics can flatten the energy landscape of the crystal structure and thereby improve the ionic conductivity. Additionally, the size of the CH_3_NH_2_ ligand and the dynamics of the CH_3_ group allow an open and flexible structure, which is also crucial for flattening of the energy landscape with respect to interstitial cationic mobility. The combined investigation of the dynamics and the ionic conductivity confirms that the compound exhibits rapid reorientations simultaneously with high ionic conductivity, similar to other metal hydridoborates.

The ionic conductivities of the three selectively deuterated samples are slightly different, see Figure 7. The fully protonated sample, Mg(BH_4_)_2_·CH_3_NH_2_, has an activation energy of 0.75 eV (72 kJ mol^–1^), consistent with previous reports.^4^ The two partly deuterated samples have slightly higher activation energies of 1.06 eV (102 kJ mol^–1^) in Mg(BD_4_)_2_·CH_3_NH_2_ and 1.00 eV (97 kJ mol^–1^) in Mg(BD_4_)_2_·CH_3_ND_2_, see Figure S10. Notably, the evolution of the ionic conductivity as a function of temperature is not completely linear, which is ascribed to a change in the pellet size during heating. Non-Arrhenius ionic conductivity has been observed for other hydridoborate-based solid electrolytes, typically closo-borate-based compounds such as Na(B_12_H_12_)_0.5_(B_10_H_10_)_0.5_.^35^ In the recent computational study of Na(B_12_H_12_)_0.5_(B_10_H_10_)_0.5_ presented in ref.,^35^ this behavior was attributed to a complex temperature-dependent change in both anion dynamics, cation disordering, and ion–ion interactions. While a similar complex behavior is also likely for Mg(BH_4_)_2_·CH_3_NH_2_ at lower temperatures, the deviation from a linear ionic conductivity behavior in the temperature range presented here is likely dominated by a change in pellet size due to the high malleability of the material. Consequently, the softness of the samples artificially increases the activation energies, as the ionic conductivities are determined from the final shape of the pellet. This leads to high uncertainties and makes it challenging to draw significant conclusions about the effect of the deuteration on the ionic conductivity.

## 4 Conclusions

The reorientational dynamics of monomethylamine magnesium borohydride (Mg(BH_4_)_2_·CH_3_NH_2_) was investigated by using quasielastic and inelastic neutron scattering (QENS and INS) combined with density functional theory calculations. The QENS results revealed that the reorientational mobility of the BH_4_^–^ anion and the CH_3_ group is rapid, while the NH_2_ group is frozen on the instrument time scale. The rapid reorentations of the BH_4_^–^ anion persist to low temperatures with a relaxation time of about ∼1–10 ns at 200 K. The dynamics of the CH_3_ groups remains rapid down to about 70 K where it has a relaxation time of about ∼1–10 ns, and the average activation energy of the reorientations was determined to be 69 ± 4 meV. The dynamics determined from the QENS data are in line with the experimental and simulated INS spectra, as well as molecular dynamics and climbing nudged elastic band calculations. The INS data revealed a flexible structure with substantial dynamics of both the BH_4_^–^ anion and the CH_3_NH_2_ ligand, and a broad band around ∼15–20 meV confirmed a reorientational activation barrier of 50–80 meV for the CH_3_ group. Molecular dynamics revealed hydrogen mean square displacements of 0.387 Å^2^ for NH_2_, 1.503 Å^2^ for CH_3_, and 1.856 Å^2^ for BH_4_^–^, and the nudged elastic band calculations showed reorientational activation energies of ∼70 and ∼74 meV for BH_4_^–^ and CH_3_, respectively.

The quasielastic neutron scattering results also suggest that the bridging BH_4_^–^ anions perform 2-fold reorientations around the *C*_2_ axis and the terminal BH_4_^–^ anions perform 3-fold reorientations around the *C*_3_ axis, while the CH_3_ group performs 3- or 6-fold reorientation around its *C*_3_ axis. Here, we show that all BH_4_^–^ anions are dynamically active already at 250 K as compared to Mg(BH_4_)_2_ where only a small fraction of the BH_4_^–^ anions is active at this temperature.^36^ The increased amount of dynamically active, rapidly reorientating BH_4_^–^ anions is likely part of the explanation for the drastic increase in ionic conductivity compared to Mg(BH_4_)_2_. While we have confirmed that high ionic conductivity and rapid reorientations happen simultaneously, further computational studies are needed to understand the direct influence of the dynamics on the ionic conductivity. This study, as one of few experimental studies on dynamics in metal borohydride-based solid electrolytes, can function as an experimental foundation for future computational studies.

## Supporting Information Available

Contains XRD and NMR data as well as additional information about the computational energy barriers, the ionic conductivity data, time-domain fits of the QENS spectra for Mg(BD_4_)_2_·CH_3_NH_2_, and mathematical expressions of relevant EISF models (PDF)
