# Supplementary material for: The Influence of Reorientational and Vibrational Dynamics on the Mg2+ Conductivity in Mg(BH4)2·CH3NH2
Source: Chem Mater. 2024 Sep 16;36(19):9784–92. doi: 10.1021/acs.chemmater.4c01947 (PMC11467906; doi:10.1021/acs.chemmater.4c01947)
Supplement: Supplementary file 1 — cm4c01947_si_001.pdf [file cm4c01947_si_001.pdf]

SUPPORTING INFORMATION

The influence of reorientational and vibrational dynamics on the Mg^{2+} conductivity in $Mg(BH_4)_2 \cdot CH_3NH_2$

Mads B. Amdisen,[1,*] Yongqiang Cheng,[2] Niina Jalarvo,[2] Daniel Pajerowski,[2]
Craig M. Brown,[3] Torben R. Jensen,[1] and Mikael S. Andersson[4,*]

[1]*Interdisciplinary Nanoscience Center (iNANO) and Department of Chemistry, University of Århus, Langelandsgade 140, DK-8000 Århus C, Denmark*

[2]*Neutron Scattering Division, Oak Ridge National Laboratory, Oak Ridge, Tennessee 37831, United States*

[3]*NIST Center for Neutron Research, National Institute of Standards and Technology, Gaithersburg, MD 20899, United States*

[4]*Department of Chemistry - Ångström Laboratory, Uppsala University, Box 538, SE-751 21 Uppsala, Sweden*

[*] M. B. Amdisen: mba@inano.au.dk

[*] M. S. Andersson: mikael.andersson@kemi.uu.se

I. POWDER X-RAY DIFFRACTION

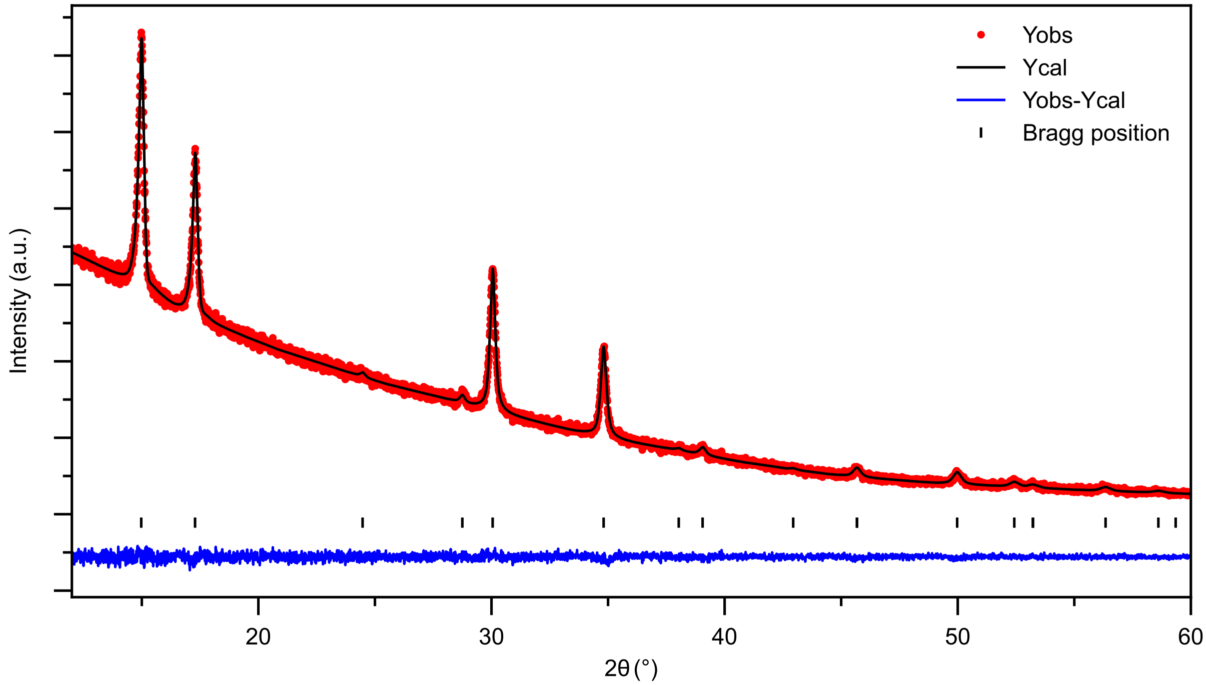

Figure S1. Rietveld refinement of in-house powder X-ray diffraction (PXD) data collected for $Mg(BD_4)_2 \cdot 6CH_3ND_2$ (λ = 1.7902 Å), showing experimental (red dots) and calculated (black line) PXD patterns, and a difference plot below (blue line). The diffraction pattern of $Mg(BD_4)_2 \cdot 6CH_3ND_2$ was collected at room temperature. Tick marks: $Mg(BD_4)_2 \cdot 6CH_3ND_2$ (100%). Final discrepancy factors: R_p = 1.93%, R_{wp} = 2.05% (not corrected for background), R_p = 34.5%, R_{wp} = 11.5% (conventional Rietveld R-factors), $R_{Bragg}(Mg(BD_4)_2 \cdot 6CH_3ND_2)$ = 0.743%, and global χ^2 = 1140.

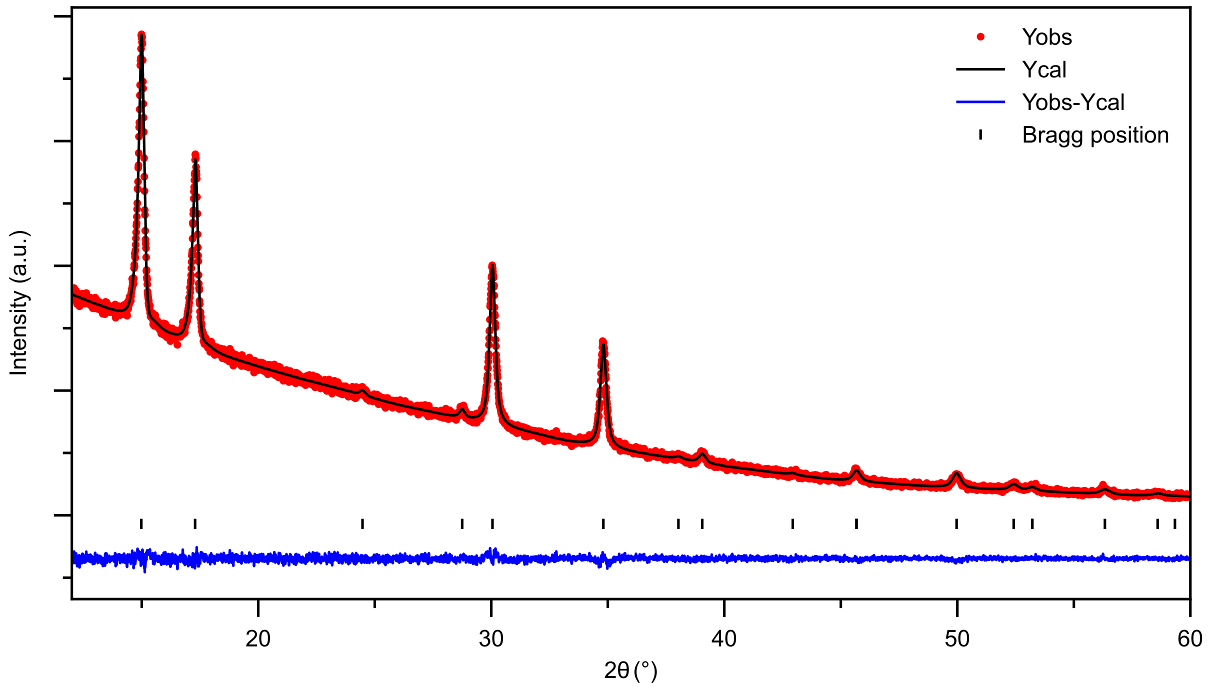

Figure S2. Rietveld refinement of in-house powder X-ray diffraction (PXD) data collected for $Mg(BD_4)_2 \cdot 6CH_3NH_2$ (λ = 1.7902 Å), showing experimental (red dots) and calculated (black line) PXD patterns, and a difference plot below (blue line). The diffraction pattern of $Mg(BD_4)_2 \cdot 6CH_3NH_2$ was collected at room temperature. Tick marks: $Mg(BD_4)_2 \cdot 6CH_3NH_2$ (100%). Final discrepancy factors: $R_p = 1.86\%$, $R_{wp} = 2.00\%$ (not corrected for background), $R_p = 24.0\%$, $R_{wp} = 8.43\%$ (conventional Rietveld R-factors), $R_{Bragg}(Mg(BD_4)_2 \cdot 6CH_3NH_2) = 0.965\%$, and global $\chi^2 = 1290$.

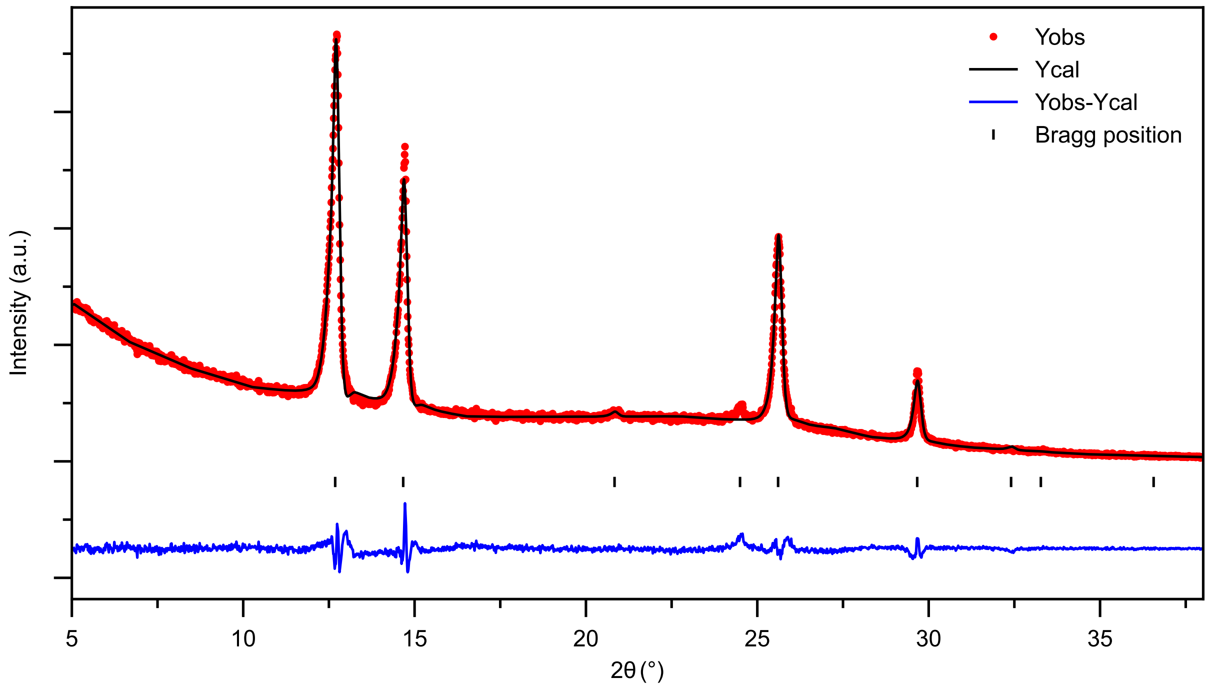

Figure S3. Rietveld refinement of in-house powder X-ray diffraction (PXD) data collected for $Mg(BH_4)_2 \cdot 6CH_3NH_2$ (λ = 1.5406 Å), showing experimental (red dots) and calculated (black line) PXD patterns, and a difference plot below (blue line). The diffraction pattern of $Mg(BH_4)_2 \cdot 6CH_3NH_2$ was collected at room temperature. Tick marks: $Mg(BH_4)_2 \cdot 6CH_3NH_2$ (100%). Final discrepancy factors: $R_p = 4.44\%$, $R_{wp} = 5.52\%$ (not corrected for background), $R_p = 21.2\%$, $R_{wp} = 9.53\%$ (conventional Rietveld R-factors), $R_{Bragg}(Mg(BD_4)_2 \cdot 6CH_3NH_2) = 1.71\%$, and global $\chi^2 = 1050$.

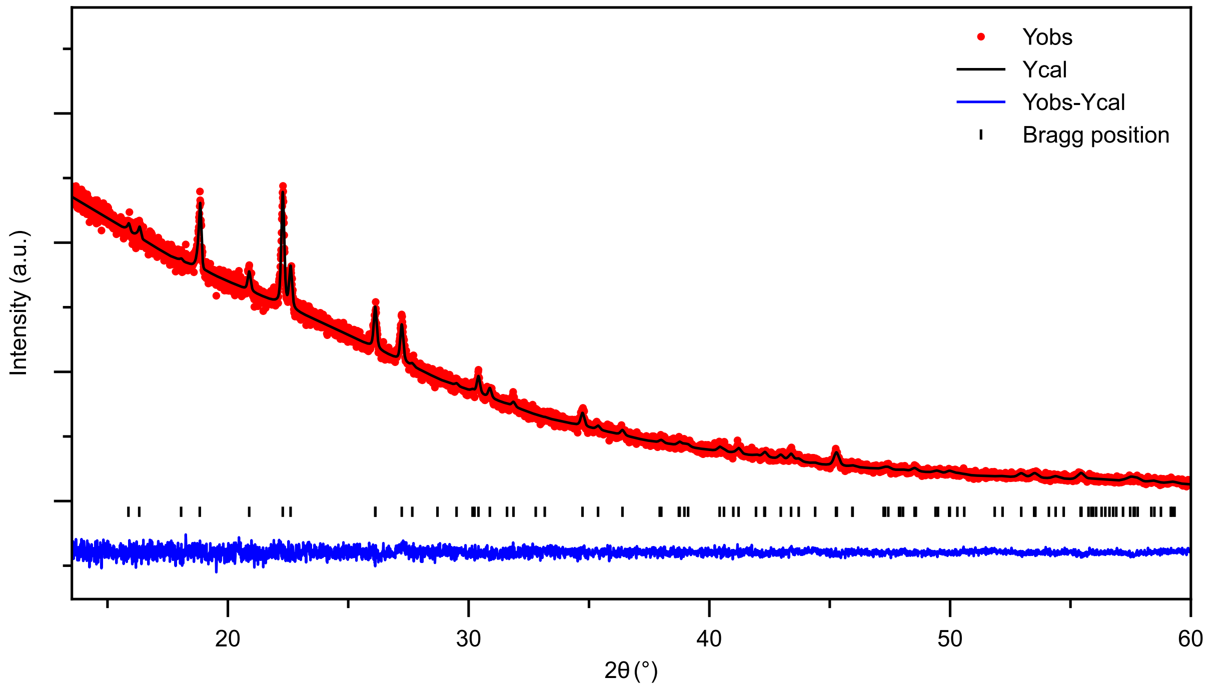

Figure S4. Rietveld refinement of in-house powder X-ray diffraction (PXD) data collected for $Mg(BD_4)_2 \cdot CH_3ND_2$ (λ = 1.7902 Å), showing experimental (red dots) and calculated (black line) PXD patterns, and a difference plot below (blue line). The diffraction pattern of $Mg(BD_4)_2 \cdot CH_3ND_2$ was collected at room temperature. Tick marks: $Mg(BD_4)_2 \cdot CH_3ND_2$ (100%). Final discrepancy factors: R_p = 2.49%, R_{wp} = 2.61% (not corrected for background), R_p = 106%, R_{wp} = 42% (conventional Rietveld R-factors), $R_{Bragg}(Mg(BD_4)_2 \cdot CH_3ND_2)$ = 14.1%, and global χ^2 = 733.

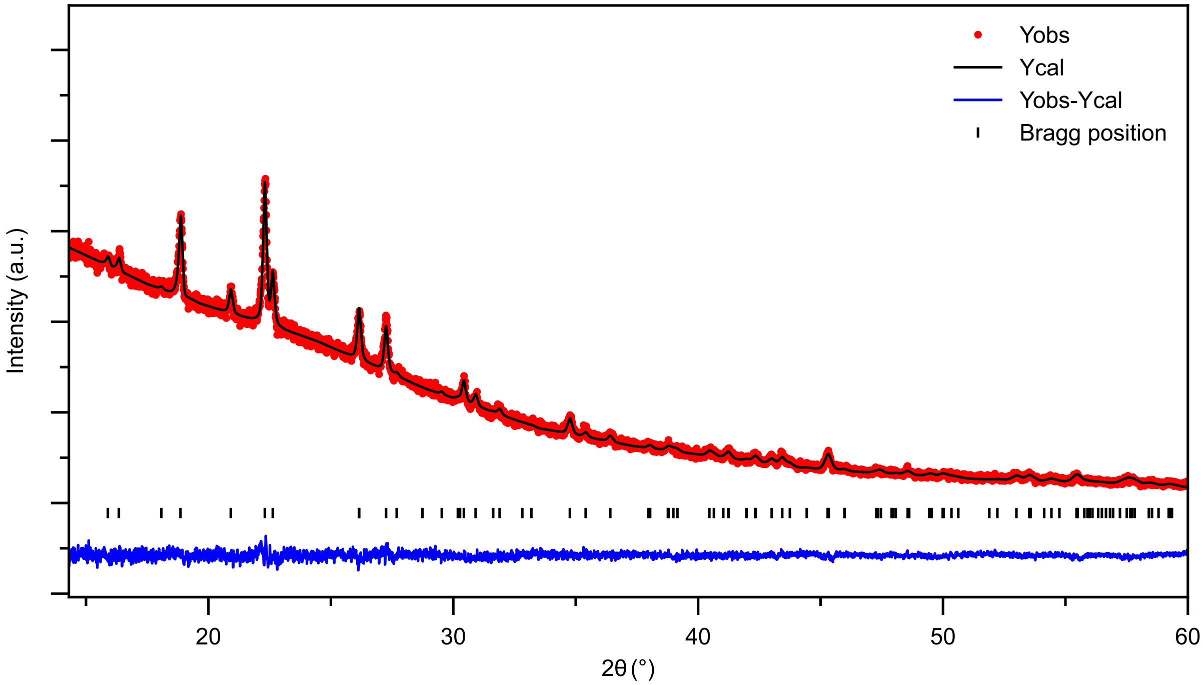

Figure S5. Rietveld refinement of in-house powder X-ray diffraction (PXD) data collected for $Mg(BD_4)_2 \cdot CH_3NH_2$ (λ = 1.7902 Å), showing experimental (red dots) and calculated (black line) PXD patterns, and a difference plot below (blue line). The diffraction pattern of $Mg(BD_4)_2 \cdot CH_3NH_2$ was collected at room temperature. Tick marks: $Mg(BD_4)_2 \cdot CH_3NH_2$ (100%). Final discrepancy factors: $R_p = 2.33\%$, $R_{wp} = 2.45\%$ (not corrected for background), $R_p = 56.5\%$, $R_{wp} = 26.8\%$ (conventional Rietveld R-factors), $R_{Bragg}(Mg(BD_4)_2 \cdot CH_3NH_2) = 13.2\%$, and global $\chi^2 = 1040$.

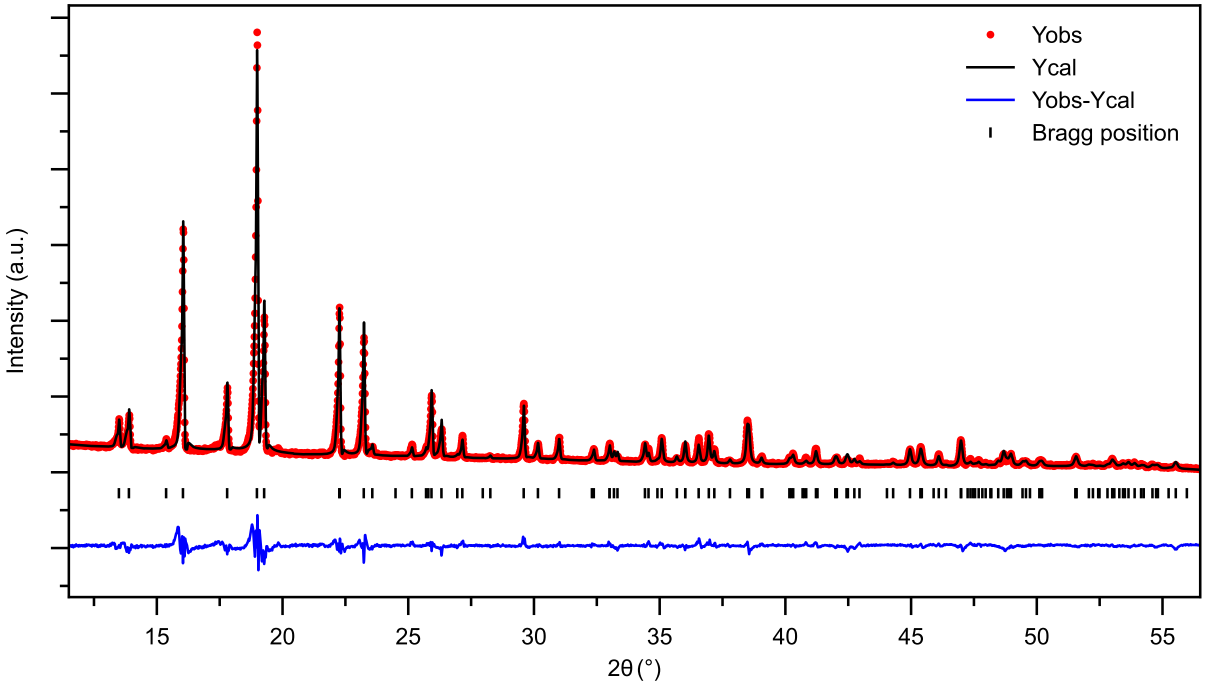

Figure S6. Rietveld refinement of in-house powder X-ray diffraction (PXD) data collected for $Mg(BH_4)_2 \cdot CH_3NH_2$ (λ = 1.5406 Å), showing experimental (red dots) and calculated (black line) PXD patterns, and a difference plot below (blue line). The diffraction pattern of $Mg(BH_4)_2 \cdot CH_3NH_2$ was collected at room temperature. Tick marks: $Mg(BH_4)_2 \cdot CH_3NH_2$ (100%). Final discrepancy factors: $R_p = 7.02\%$, $R_{wp} = 8.58\%$ (not corrected for background), $R_p = 20.8\%$, $R_{wp} = 11.5\%$ (conventional Rietveld R-factors), $R_{Bragg}(Mg(BH_4)_2 \cdot CH_3NH_2) = 7.12\%$, and global $\chi^2 = 4510$.

II. NUCLEAR MAGNETIC RESONANCE SPECTROSCOPY

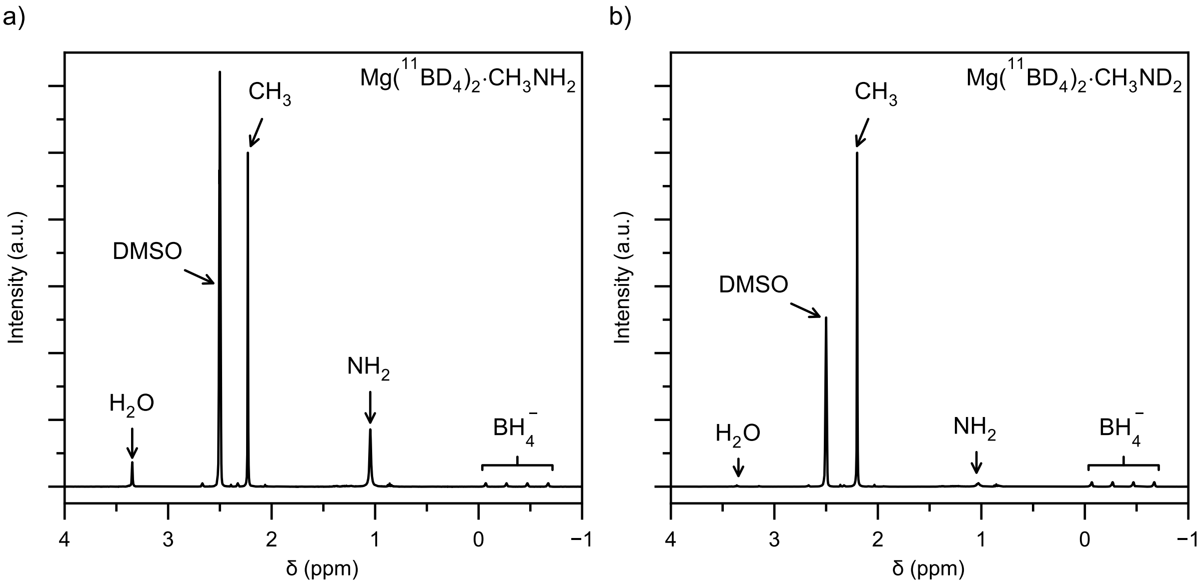

Figure S7. ^{1}H NMR spectra of a) $Mg(^{11}BD_4)_2{\cdot}CH_3NH_2$, and b) $Mg(^{11}BD_4)_2{\cdot}CH_3ND_2$.

Table S1. ^{1}H NMR integrated intensities (I) for each moiety, i.e. CH_3, NH_2, and BH_4^-, normalized to the integrated intensity of the CH_3 moiety. The number of protons per moiety (H/mo.) is also provided as well as the relative percentage of protons for each moiety (H pct.).

	$Mg(^{11}BD_4)_2{\cdot}CH_3NH_2$			$Mg(^{11}BD_4)_2{\cdot}CH_3ND_2$		
Moiety	I	H/mo.	H pct. (%)	I	H/mo.	H pct. (%)
CH_3	1.00	3	100	1.00	3	100
NH_2	0.67	2	100	0.07	0.21	10.5
BH_4^-	0.11	0.17	4.1	0.15	0.23	5.6

III. SYNCHROTRON RADIATION POWDER X-RAY DIFFRACTION

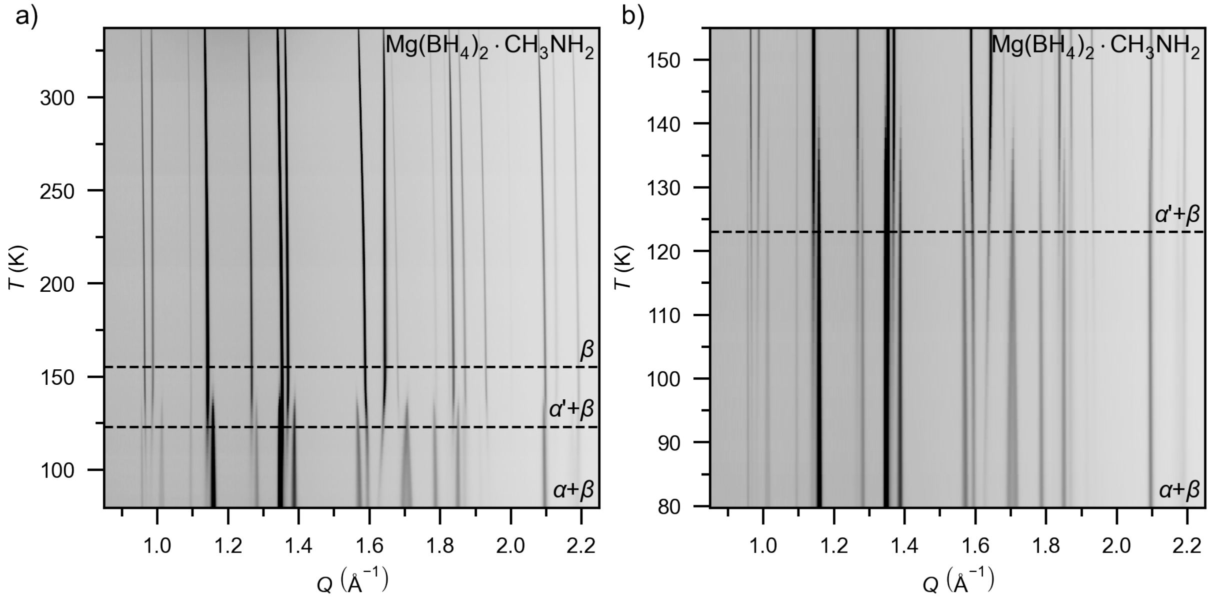

Figure S8. *In situ* synchrotron radiation powder X-ray diffraction data of $Mg(BH_4)_2 \cdot CH_3NH_2$ in the temperature ranges a) 80-335 K and b) 80-155 K.

IV. COMPUTATIONAL INVESTIGATIONS

Table S2. Density functional theory optimized unit cell parameters of α-$Mg(BH_4)_2 \cdot CH_3NH_2$.

Unit cell parameter	Value
Space group	$P1$
a (Å)	8.0014
b (Å)	14.7696
c (Å)	11.4915
α (°)	89.7720
β (°)	90.0080
γ (°)	89.7450
V (Å^3)	1358.01
Z	8
V/Z (Å^3)	169.751
M (g/mol)	85.036
ρ (g/mL)	0.83186

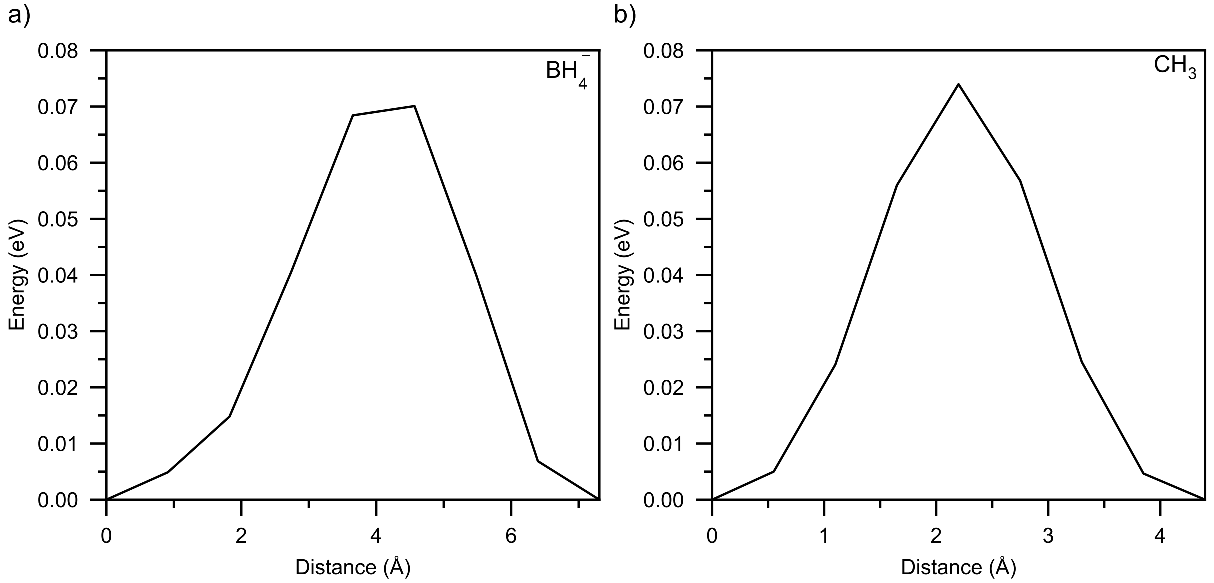

Figure S9. Rotation barriers determined with the climbing image nudged elastic band method of a) BH_4^-, and b) CH_3.

V. IONIC CONDUCTIVITY

Magnesium ionic conductivity data were fitted by linear regression in $\ln(\sigma T)$ vs. T^{-1} plots. The activation energy of each compound was calculated from a modified Arrhenius equation:

$$\sigma = \frac{\sigma_0}{T} \cdot e^{\frac{E_A}{k_B T}} \tag{1}$$

where σ is the Mg^{2+} ionic conductivity, T is the temperature, σ_0 is a pre-factor, E_A is the activation energy, and k_B is the Boltzmann constant. The linear regressions are shown as dark red lines in Fig. S10.

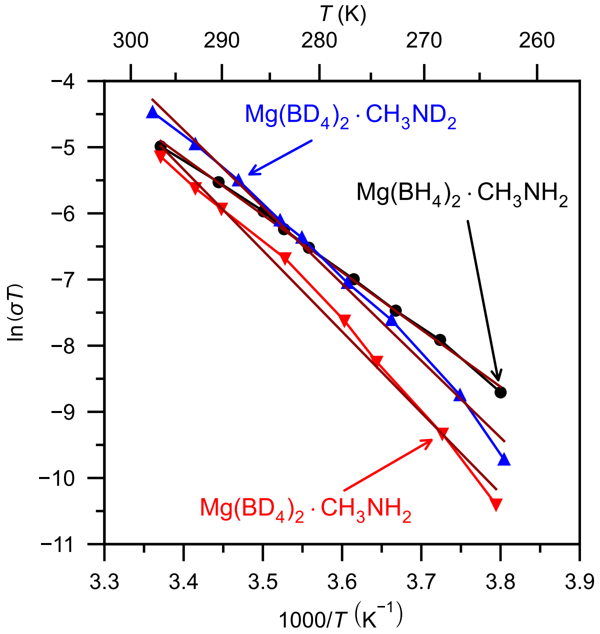

Figure S10. Linear fits (dark red lines) of ionic conductivity data on $Mg(BH_4)_2{\cdot}CH_3NH_2$ (black, circles), $Mg(BD_4)_2{\cdot}CH_3NH_2$ (red, down triangles), and $Mg(BD_4)_2{\cdot}CH_3ND_2$ (blue, up triangles).

VI. QUASIELASTIC NEUTRON SCATTERING

A. *I(Q,t)*

Figure S11 shows QENS data from CNCS collected using a neutron energy of 2.49 meV in the temperature range 100 K to 295 K, which has been fast Fourier transformed from $S(Q,\omega)$ to $I(Q,t)$, i.e. from energy domain into time domain. The figure also shows fits to this data using the KWW function, $I = I_0 e^{(t/<\tau>)^\beta}$, where t is the time, I the intensity, I_0 the intensity at $t = 0$, $< \tau >$ the average relaxation time of the distribution and β the stretching exponent, which is related to the distribution of relaxation times.

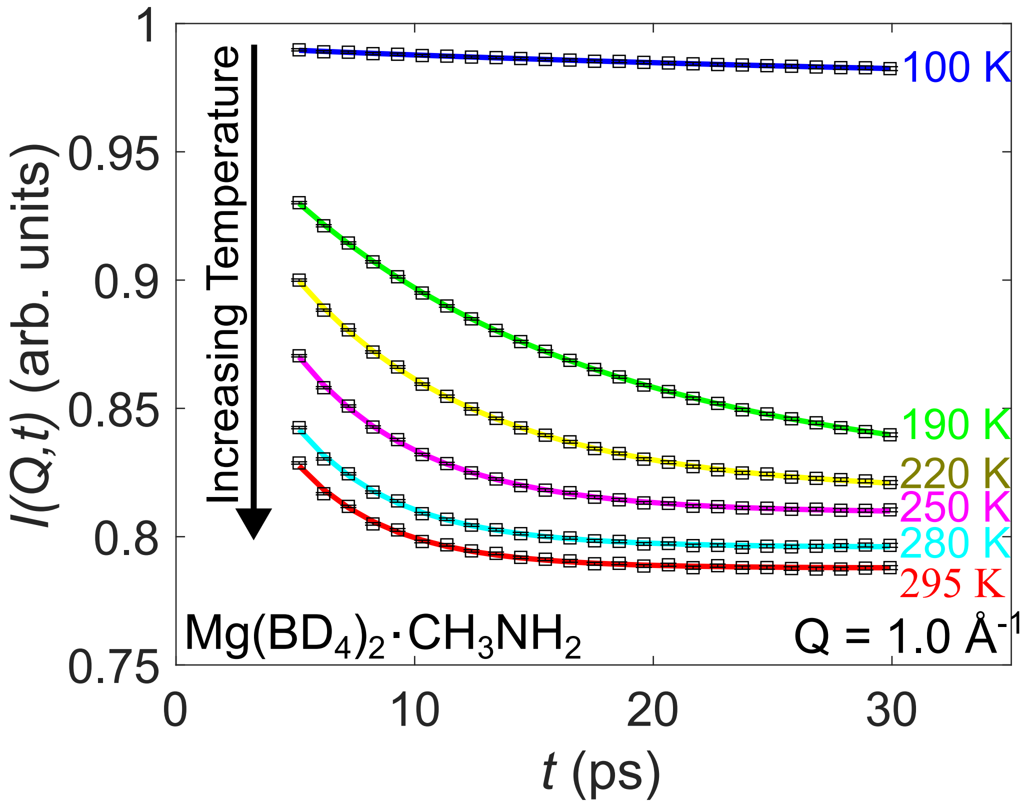

Figure S11. $I(Q,t)$ data (black squares) in the temperature range 100 K to 295 K for $Mg(BD_4)_2{\cdot}CH_3ND_2$ with fits (solid lines) to $I = I_0 e^{(t/<\tau>)^\beta}$ using $\beta = 0.94$. The error bars correspond to 1 standard deviation.

B. Q-dependence of the quasielastic broadening

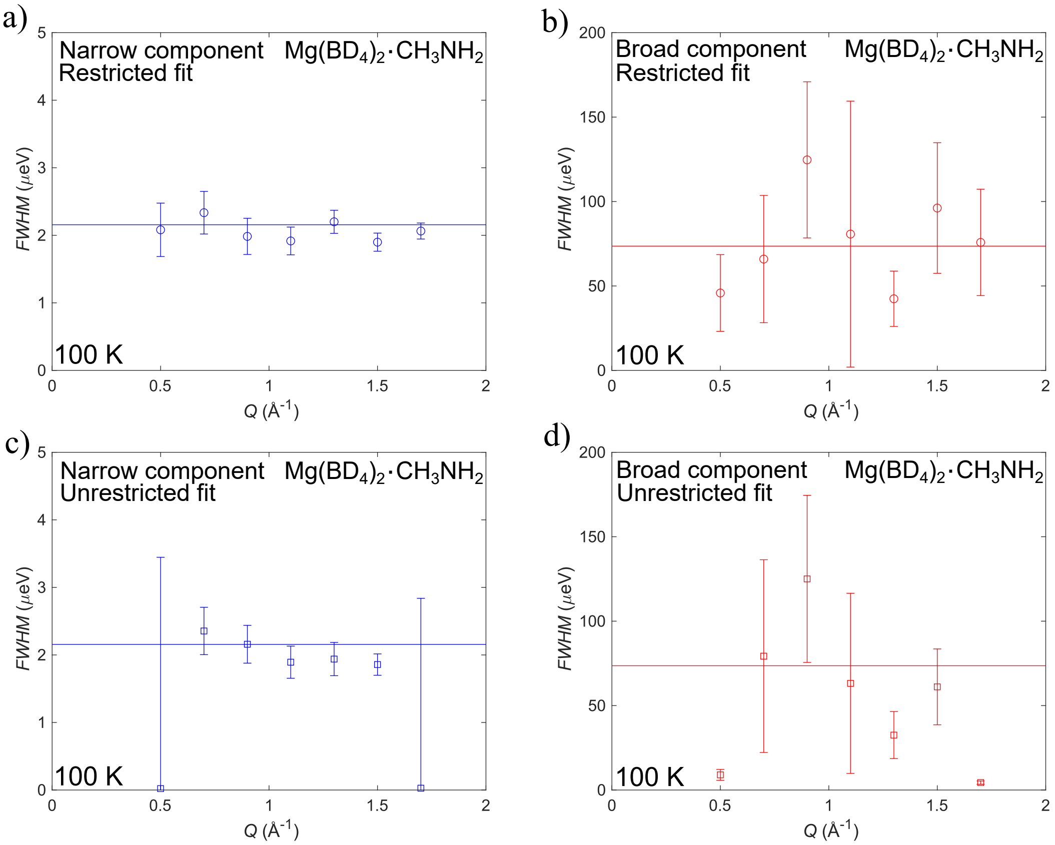

Figure S12. Q-dependence of the quasielastic broadening for $Mg(BD_4)_2{\cdot}CH_3NH_2$ at 100 K collected using BASIS (Si111 analyzer). The QENS broadening in this energy range can be accurately described using 2 Lorentzians, one narrow and one broad. The solid lines represent the quasielastic broadening extracted from a fit of the data using a single Q-bin (Q=0.3 to 1.7Å^{-1}). In a) and b) the broadening of one component was extracted, while keeping the other fixed to the value determined from the single Q-bin fit, i.e. in a) the narrow component was determined while keeping the broad component fixed. In c) and d) both components were allowed to vary simultaneously. The error bars correspond to one standard deviation and the Q-bin size is 0.2 Å^{-1}.

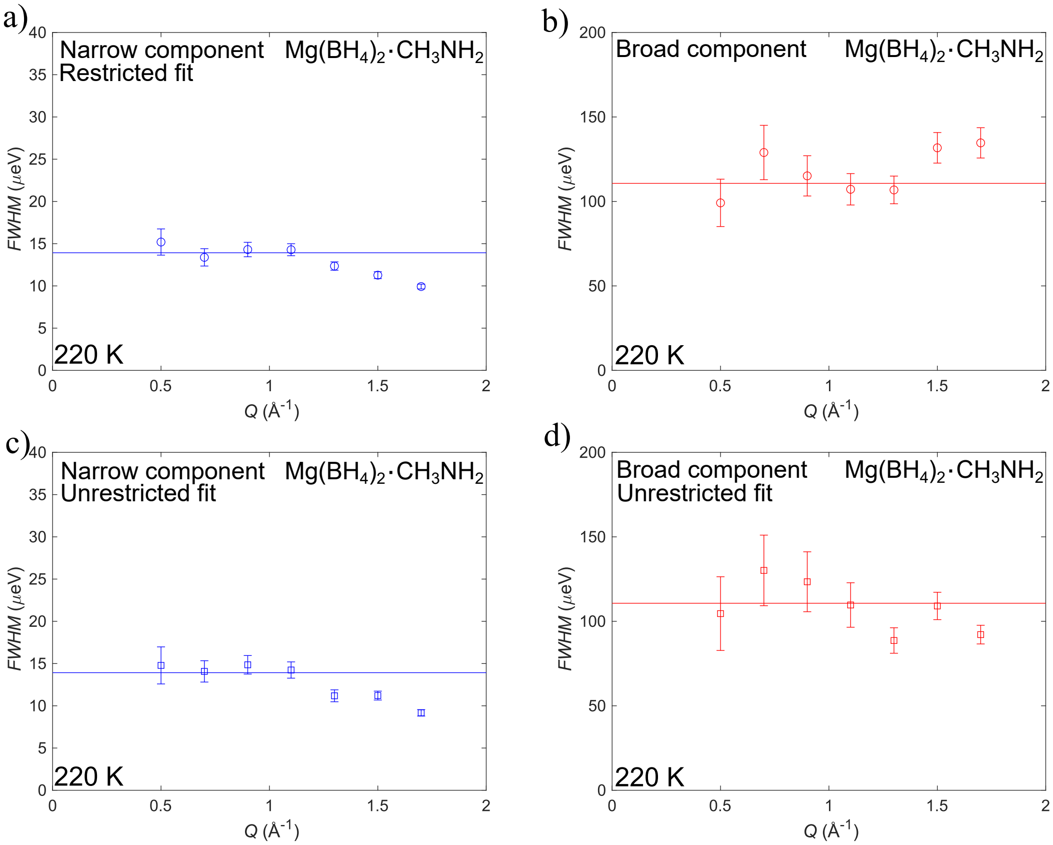

Figure S13. Q-dependence of the quasielastic broadening for $Mg(BH_4)_2{\cdot}CH_3NH_2$ at 220 K collected using BASIS (Si111 analyzer). The QENS broadening in this energy range can be accurately described using 2 Lorentzians, one narrow and one broad. The solid lines represent the quasielastic broadening extracted from a fit of the data using a single Q-bin (Q=0.3 to 1.7Å^{-1}). In a) and b) the broadening of one component was extracted, while keeping the other fixed to the value determined from the single Q-bin fit, i.e. in a) the narrow component was determined while keeping the broad component fixed. In c) and d) both components were allowed to vary simultaneously. The error bars correspond to one standard deviation and the Q-bin size is 0.2 Å^{-1}.

C. Mathematical expressions of the EISF model curves for CH_3, NH_2 and BH_4^-

The EISF models for C_2 and C_3 reorientation[S1] for a tetrahedral molecule or ion such as BH_4^- are given by:

$$\mathrm{EISF}_{C_2/C_3,\,\mathrm{BH}_4^-} = \frac{1 + j_0(Qd)}{2}, \tag{2}$$

where d is the jump distance and $j_0 = \sin(x)/x$ is the zeroth-order spherical Bessel function. For a trigonal pyramidal molecule/group such as CH_3 the EISF model for C_3 and C_6 reorientation[S2] is given by:

$$\mathrm{EISF}_{C_3,\,\mathrm{CH}_3} = \frac{1 + 2j_0(Qr\sqrt{3})}{3}, \tag{3}$$

and

$$\mathrm{EISF}_{C_6,\,\mathrm{CH}_3} = \frac{1 + 2j_0(Qr) + 2j_0(Qr\sqrt{3}) + j_0(2Qr)}{6}, \tag{4}$$

where r is the radius of the circle of rotation, which for the CH_3 group of the CH_3NH_2 molecule is 1.04 Å. For a bent molecule/group such as NH_2 the EISF model for C_2 and C_4 reorientation[S2] is given by:

$$\mathrm{EISF}_{C_2,\,\mathrm{NH}_2} = \frac{1 + j_0(2Qr)}{2}, \tag{5}$$

and

$$\mathrm{EISF}_{C_4,\,\mathrm{NH}_2} = \frac{1 + 2j_0(Qr\sqrt{2}) + j_0(2Qr)}{4}. \tag{6}$$

For the NH_2 group of the CH_3NH_2 molecule r is 0.80 Å. The EISF = 1 for a molecule/group/ion which is frozen on the experimental time scale. To form the total EISF of the system each EISF has to be weighted by its corresponding fraction of the the total incoherent scatting of the sample. For $Mg(BH_4)_2{\cdot}CH_3NH_2$ and its corresponding deuterides the incoherent scattering of ^{11}B, C and N are very small and thus do not have to be taken into account when calculation the fractions for each EISF.

References

S1. Yildirim, T.; Gehring, P. M.; Neumann, D. A.; Eaton, P. E.; Emrick, T. Neutron-scattering investigation of molecular reorientations in solid cubane. *Phys. Rev. B* **1999**, *60*, 314–321

S2. Bée, M. *Quasielastic Neutron Scattering*; Adam Hilger, Bristol, 1988
